# Charitable Food Systems’ Capacity to Address Food Insecurity: An Australian Capital City Audit

**DOI:** 10.3390/ijerph15061249

**Published:** 2018-06-12

**Authors:** Christina M. Pollard, Bruce Mackintosh, Cathy Campbell, Deborah Kerr, Andrea Begley, Jonine Jancey, Martin Caraher, Joel Berg, Sue Booth

**Affiliations:** 1 Faculty of Health Science, School of Public Health, Curtin University, GPO Box U1987, Perth 6845, Australia; Cathy.Campbell555@gmail.com (C.C.); D.Kerr@curtin.edu.au (D.K.); A.Begley@curtin.edu.au (A.B.); J.Jancey@curtin.edu.au (J.J.); 2 School of Agriculture and Environment, The University of Western Australia, 35 Stirling Highway, Crawley, Perth 6009, Australia; bruce.mackintosh@uwa.edu.au; 3 Centre for Food Policy, City University of London, Northampton Square, London EC1V 0HB, UK; m.caraher@city.ac.uk; 4 Hunger Free America, 50 Broad Street, Suite 1103, New York 10004, NY, USA; JBerg@hungerfreeamerica.org; 5 College of Medicine & Public Health, Flinders University, GPO Box 2100, Adelaide 5000, Australia; sue.booth@flinders.edu.au

**Keywords:** food insecurity, charitable food services, food charity, food system, nutrition, voluntary failure

## Abstract

Australian efforts to address food insecurity are delivered by a charitable food system (CFS) which fails to meet demand. The scope and nature of the CFS is unknown. This study audits the organisational capacity of the CFS within the 10.9 square kilometres of inner-city Perth, Western Australia. A desktop analysis of services and 12 face-to-face interviews with representatives from CFS organisations was conducted. All CFS organisations were not-for–profit and guided by humanitarian or faith-based values. The CFS comprised three indirect services (IS) sourcing, banking and/or distributing food to 15 direct services (DS) providing food to recipients. DS offered 30 different food services at 34 locations feeding over 5670 people/week via 16 models including mobile and seated meals, food parcels, supermarket vouchers, and food pantries. Volunteer to paid staff ratios were 33:1 (DS) and 19:1 (IS). System-wide, food was mainly donated and most funding was philanthropic. Only three organisations received government funds. No organisation had a nutrition policy. The organisational capacity of the CFS was precarious due to unreliable, insufficient and inappropriate financial, human and food resources and structures. System-wide reforms are needed to ensure adequate and appropriate food relief for Australians experiencing food insecurity.

## 1. Introduction

The health consequences of socio-economic disadvantage, including homelessness, are increasingly seen in high income countries, including Australia [1,2,3,4]. Food insufficiency is closely associated with poor mental and physical health [5,6,7,8] and is common among people who are homeless [5,9,10].

Cities attract vulnerable populations experiencing food insecurity as they provide concentrated food and support services. The types of people accessing inner-city charitable food services (CFS) are highly variable and include people who are homeless or domiciled and in financial difficulty due to a range of circumstances, those living in hostel and shelters, backpackers, and women fleeing from domestic violence. The inner-city precinct of Perth, the capital city of Western Australia, covers 10.9 square kilometres with a population of about 32,000 people. The Perth Homeless Registry showed that the number of street-present people increased from 192 to 319 between 2012 and 2016 [11]. Over 80% had been homeless for six months or more and 52% had complex comorbidities of existing medical conditions, mental illness and substance use disorders [11].

Concerns have been raised regarding the ongoing capacity of food relief systems in both Australia and the United States (U.S.) to meet the increasing demand [12,13]. There is also evidence of sub-optimal nutritional quality in the food provided [14,15]. Australia’s response to food insecurity is at a critical point given the increasing need, the absence of government-funded food assistance such as in the U.S. [15] and the diminishing welfare safety net [16]. Australian Government welfare policies designed to reduce poverty—for example, the Australian Age Pension [17], Family Tax Benefit and the Child Care Subsidy [18] and Newstart Allowance [19]—provide assistance to low income earners and may assist in improving food security; however, the evidence for the increasing demand for food relief suggests they are inadequate.

Food charity, the delivery of donated, unsaleable, or waste food by the voluntary (non-profit) sector, is the dominant response to food insecurity in Australia [12]. This charitable food system (CFS), originally designed to provide immediate short-term food relief, is struggling as food insecurity and the demand for food assistance is chronic and increasing [12,20]. In 2015, there were 3000 to 4000 food relief services nationally [12]. The demand for food relief services increased in 2016, up 8% from 2015 [21].

Although short-term need is assumed in Australia [22], there is evidence of long-term reliance on the CFS [23,24]. The Australian response to food insecurity has been described as ad hoc with numerous small voluntary organisations providing food assistance [25]. Internationally, researchers have questioned whether the expansion and reliance on food charity and food banking is the appropriate response to food insecurity in developed countries, based on users’ negative experiences of shame and being stigmatised and poor quality or limited food choices [26]. The CFS consists of both “in-direct” services (IS) (food banking and rescue organisations who collect, bank and/or distribute unsaleable food) and ‘direct’ services (DS) who provide the food to those in need.

The non-profit (NP) sector’s ability to effectively address problems such as food insecurity has also been questioned in the academic literature, which has been critical of food banks based on users’ experiences such as shame, stigma and eligibility criteria in high-income countries [20,27]. Salamon’s theory of voluntary failure was developed in 1987 to explain the effectiveness of the voluntary response to issues such as food insecurity [28]. His theory described market failure, voluntary failure, and third-party government failure in delivering effective welfare-government relationships in the United States. The CFS NP voluntary response in Australia has arisen as a result of both a market and a third-party government failure in delivering the “collective good” of providing a welfare safety net to prevent food insecurity. The Commonwealth Department of Social Services acknowledges the existence of Emergency Relief as “services delivered by community organisations”.

Salamon’s four types of voluntary failure include (i) philanthropic insufficiency, the “inability to generate resources on a scale that is both adequate enough and reliable enough to cope with the human-service problems” [28] (p. 39); (ii) philanthropic particularism, which occurs when “some subgroups of the community may not be adequately represented in the structure of voluntary organizations” [28] (p. 40) where the focus is on treating “the more ‘deserving’ of the poor” leaving serious service gaps or duplicating services and wasting resources; (iii) philanthropic paternalism, which refers to the notion that “those with the greatest resources have influence over the definition of community need” [28] (p. 41); and (iv) philanthropic amateurism, described as “amateur approaches to coping with human problems” [28] (p. 42). A current assessment of CFS in Perth shows evidence of one type of voluntary failure, namely philanthropic insufficiency [24].

There has been limited exploration of the scope and organisational capacity of the Australian CFS [12,29] and none in Western Australia. In 2014, due to increasing demand, food relief organisations expressed a need to understand the current and future capacity of the CFS in inner-city Perth to meet their clients’ needs [25]. Understanding the practical organisational issues facing the CFS is important when considering options for change to improve end-user services [30]. The aim of this paper is to document the scope, nature and organisational capacity of the CFS located in or serving inner-city Perth.

## 2. Methods

Between July and September 2015, an organisational capacity audit was undertaken of the CFS located in or serving inner-city Perth. The audit identified and mapped component organisations, their values, human and financial resources, their networks, nutrition policies, and food service operations.

A research advisory group comprising the research team and five representatives from key organisations working with homelessness, social disadvantage and relief services identified the CFS organisations located in or serving inner-city Perth. They provided initial contact details which were confirmed via telephone or web search. A nomenclature for the types of food service models and their inter-relationships was agreed, for example IS and DS.

Two semi-structured interview schedules were developed for the IS and DS. The assessment of organisational capacity was guided by the approach used to improve nutrition for vulnerable groups, children in childcare in Australia [31] and food bank users in the United States [15]. The instrument was adapted from the Research Tools for Use in Studying Nutrition Policies and Practices in the Emergency Food Bank Network [15] and Food Service Planning in Child Care in Western Australia [32] surveys, which assessed organisational capacity for a safe, nutritious and appropriate food service. Table 1 provides an overview of the interview schedule.

The surveys were trialed with senior managers from two CFS organisations, with changes made to the order and phrasing of questions post-pilot to ensure clarity. Background information was obtained from organisational websites, and interviewees were asked to provide written documentation such as annual reports or service brochures.

Two researchers (Bruce Mackintosh and Cathy Campbell) with extensive experience in food relief and public health nutrition conducted the interviews. Thirty DS and five IS were telephoned to screen for interview suitability. If the organisation played a role in food service delivery in inner-city Perth, the chief executive officer, director or manager, or a nominated proxy was invited for face-to-face interview. Twelve face-to-face interviews were conducted (nine DS and three IS). Eight telephone interviews were conducted with DS offering limited food relief; for example, only supermarket vouchers or one-off cash payments to their clients. Both researchers attended each interview, filled in the written questionnaire and took additional notes. The three lots of data—the audit of the websites, interviewee responses to the survey instrument and interviewer notes—were collated, reported in tables where appropriate, and general findings were summarised by the interviewers. The study was conducted according to guidelines in the Declaration of Helsinki and all procedures involving human subjects were approved by the Curtin University Human Research Ethics Committee (HR183/2015). Written informed consent was obtained from all subjects.

## 3. Results

This study is the first to describe the organisational capacity of the CFS located in or servicing a capital city in Australia. The inner-city Perth CFS comprised three indirect services (IS) who sourced, banked and/or distributed food to 15 direct services (DS), who in turn provided food to recipients. DS offered 30 different food services at 34 locations feeding over 5670 people/week via 16 models including mobile and seated meals, food parcels, supermarket vouchers, and food pantries: see Table 1.

**Figure 1:**
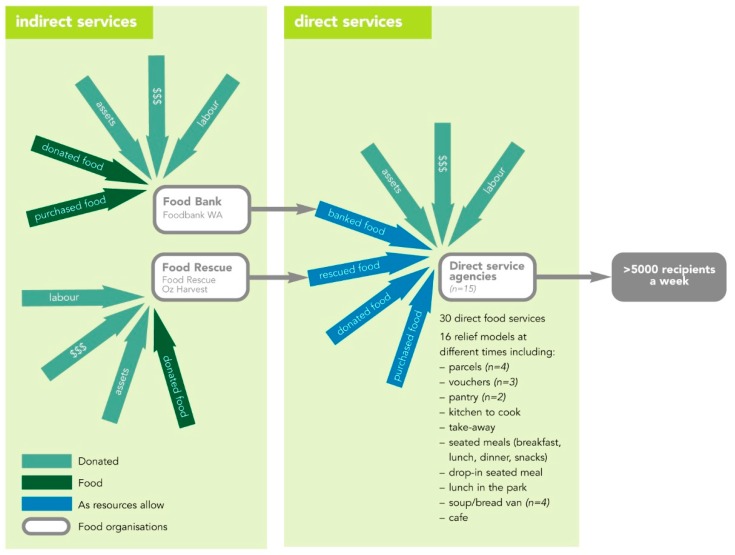
Model of the charitable food sector in inner-city Perth.

The CFS organisational capacity is described in terms of purpose; years of operation; funding sources and workforce structure; food supply and food service models offered; commitment to and structures to support the provision of nutritious food; the influence/impact of government regulation or legislation. The organisational overview of the CFS serving inner-City Perth is shown in Figure 1, and the audit results described for IS and then for DS followed by the barriers to improvement and interviewee recommendations.

### 3.1. Indirect Services

Three IS were either located in or serviced inner-city Perth at the time of the research who procured food and either banked, sorted or directly distributed it to DS; Supplementary Table S1 shows the characteristics of the IS.

#### 3.1.1. Organisational Intent, Funding and Workforce

Foodbank WA (Perth Western Australia), had operated for 21 years in, compared to 2 and 5 years for OzHarvest and Food Rescue WA, respectively. All three IS aimed to rescue surplus food and reduce food waste to provide for people in need, either via DS or directly. Foodbank WA and Food Rescue WA included reducing hunger with nutritious food and Foodbank WA and OzHarvest mentioned quality food. Food Rescue WA merged with Uniting Care West, a community services agency of the Uniting Church in Australia Synod of WA in 2013.

IS managers said their funding was ad hoc, unreliable, and from different sources including corporate and private donations, sponsorships and government grants. Foodbank WA also charged DS a per-kilogram handling fee to cover operating costs which generated AUD$3.78 million in 2017 [33]. OzHarvest and Food Rescue WA relied entirely on donations.

The workforce varied with organisational size: the number of paid full-time equivalent (FTE) staff ranged from two to 50 (Food Rescue WA and Foodbank WA respectively). Foodbank and OzHarvest employed nutritionists/dietitians (10 FTE and 1 casual respectively), one chef (1 FTE and 1 casual respectively) and hosted student placements. All three IS relied heavily on volunteers: Food Rescue WA had 2 paid staff and 100 volunteers (1:50), Foodbank WA has 1:45 and OzHarvest 1:25.

#### 3.1.2. Food Types, Sources, Collection and Distribution

All IS sourced donated food through partnerships with the local food and grocery industry or retailers. Foodbank WA estimated that it receives over 80% of all donated food in WA and the larger DS in inner-city Perth sourced food from them. The type of food collected varied: 74% of Foodbank WA food was packaged non-perishable; 99% of Food Rescue WA and OzHarvest’s food is perishable (e.g., fruit and vegetables, frozen meals, sushi, prepared sandwiches and quiches), collected in refrigerated vans from cafes, restaurants, supermarkets, caterers and bakeries. Overall, food donations did not meet demand and, despite an interest in discouraging unhealthy food donations, IS all accepted them.

Foodbank WA established their ‘key staples program’ (KSP) to meet the demand for healthy food. KSP is an alliance with suppliers who donate or subsidize the cost of ingredients, packaging and delivery of nine products (flour, pasta sauce, oats, spaghetti, and canned baked beans, tomatoes, fruit, vegetables and soup). Foodbank WA’s expenditure on KSP represented 8.95% of their total expenditure in 2017 [33].

#### 3.1.3. Facilities, Size and Function

IS varied in facilities, size and function. Foodbank WA’s, the largest, has premises located 27 kilometres from CBD. They operate as a food storage depot and 510 DS collect food from them, paying a small handing fee. There is substantial warehouse storage with refrigeration and freezers and a commercial kitchen. A fleet of trucks collects food from major supermarkets and they prepare 2000 meals each week onsite and freeze them to sell to DS. A small number of people, referred by DS, can purchase food from directly as well.

Food Rescue WA has premises 10 km from the CBD with refrigerated and freezer storage space where they sort and repack the food they collect in two refrigerated trucks from supermarkets, then deliver to DS. They also use ‘cargo carts’ to collect and redistribute sandwiches and wraps from cafes in the CBD each day. OzHarvest distributes the food to DS immediately.

In 2016, Foodbank WA rescued 2.8 million kg of food, OzHarvest rescued 348,627 kg and distributed it to 84 DS [34] and Food Rescue WA distributed 478,000 kg of food [33]. Most (75%) of OzHarvest’s food is rescued from supermarkets.

Overall, despite rescuing and redistributing significant quantities of food, the IS said they were unable to meet the demand and provide a sustainable, consistent supply of nutritious food to DS agencies who consequently had to make daily modifications to their services.

#### 3.1.4. Nutrition Policy and Capacity

No IS had a formal nutrition policy to specify the types of foods procured. The Foodbank WA interviewee said that the presence of the nutrition staff encouraged a preference for nutritionally preferable KSP and the amount of sugar-sweetened beverages and potato crisps they distributed had declined. Food Rescue WA mainly focused their effort on the procurement of fresh fruit and vegetables.

All paid food preparation staff at Foodbank WA were food safety trained and premises were regularly inspected by local government officers. The two food rescue organisations said they did not accept unsafe food so did not train staff in food safety. OzHarvest offered two sessions of their Nutrition Education Sustenance Training (NEST) nutrition education program for staff, volunteers and service recipients.

#### 3.1.5. Organisational Relationships

Some DS were not aware of the extent and nature of Foodbank WA’s services and capacity to supply, at no or a very small cost, many of the items they were purchasing at full price from supermarkets. Some of those who were aware did not use Foodbank WA because they lacked regular transport and the distance from the CBD was a barrier. Others who accessed Foodbank WA purchased energy dense-nutrient poor foods such as potato crisps because they were cheaper by weight than heavier nutritious foods.

### 3.2. Direct Services

#### 3.2.1. Organisational Intent, Funding and Workforce

The DS had provided food charity in inner-city Perth for many years: for example, the Salvation Army has provided food relief for 125 years while others have done so for 35 to 60 years. Ten of the 15 organisations had faith-based origins. Humanitarian values with a commitment to human dignity and a finding pathway out of food security guided most, typified by one interviewee’s comment: “We believe that by nourishing the homeless and the vulnerable in a non-judgmental and compassionate way, we give hope, raise awareness about poverty and provide better outcomes for the wider community”.

Funding sources were described as diverse and difficult to quantify. Three of the 15 DS received government funding (e.g., grants from State Government (Departments of Health or Child Protection and Family Support) or the Commonwealth Department of Social Services via their Emergency Relief Fund). Faith-based DS relied on parent organisations such as their church as well as corporate or public donations. Non-faith-based DS relied on philanthropic donations, fundraising activities, and community grants from funders such as Lotterywest, the official State lottery for WA. Specific information on corporate donors was not provided for confidentiality reasons.

The DS workforce structure is summarized in Table 2. The reliance on volunteers was high at a paid staff to volunteer ratio of 1:37. Although many interviewees said that they would value input from a nutritionist or dietitian, only two DS had access to formally trained nutrition personnel. One had employed a full-time dietitian but terminated the position in December 2015 due to lack of funds and the other dietitian was only employed on a casual basis to plan menus.

#### 3.2.2. Food Service Models, Number and Facilities

Table 2 outlines the types of food service models and number of facilities (4 mobile services, 7 with premises, 2 shelters and 4 for specific client groups, e.g., people at risk of HIV/AIDS). There were 16 food service models offered at different locations on various days in the week: mobile and seated meals food parcels and pantries, and vouchers. Most DS recipients were homeless men, with a high proportion of Aboriginal and/or Torres Strait Islander people. Weekend coverage was limited. Four vans distributed prepared food in parks: one offered a three-course lunchtime meal 5-days/week, one offered soup, pies, sandwiches, tea and coffee each morning, another soup and bread each evening, and the fourth offered sandwiches to young people on the street 5 days/week.

Seven DS with premises had food preparation and serving areas and offered seated meals, usually on weekdays in one large room. Based on an eligibility assessment of need, five provided food parcels and pantry (a small storeroom with mostly non-perishable food items arranged on shelves) visits. A staff member accompanied eligible recipients to the pantry where they choose a set number of free food items or a food parcel, deemed sufficient for 1–2 days to enable a single person or a family to “get back on their feet”. Pantry visits are restricted to once or twice a year. 

Two DS only provided vouchers or “gift cards” to purchase food from supermarkets with eligibility based on DS-assessed need. Vouchers can be redeemed for food and/or any supermarket items other than cigarettes and alcohol. Recipients often purchase non-food items: for example, toiletries or dog food. Several DS provided school breakfasts or delivered frozen meals to other agencies.

#### 3.2.3. Food Sources

Figure 1 shows DS food sources. Most DS use more than one source of food (either from IS or from direct food donations or purchased directly from supermarkets using donated money) and quantities varied from week to week. Some went to Foodbank WA for free food but chose lighter food by weight due to the handling fee while others relied on food rescue delivered daily. Church groups and philanthropic supporters intermittently offered food to DS. Most of the DS food supply was non-perishable and shelf-stable such as pasta or canned tuna, with the exception of daily rescued food. Interviewees said they needed more donations of perishable whole foods, particularly fruit and vegetables, meat, fish and dairy products.

#### 3.2.4. Nutrition Policy and Capacity

Interviewees said that DS were generally wanting to improve nutrition standards but none had a formal nutrition policy and only one listed nutrition and food safety as program priorities. Interviewees did not believe that their reliance on donated food would influence nutrition policy actions they might choose to implement, but also said that they were unwilling to refuse donations of poor nutrition quality. The increasing and unmet demand for food, uncertain and unreliable food supply, and the salience of nutrition messages among volunteers and recipients were listed as barriers to improving the healthfulness of the food DS provided. The interviewers noted the poor nutrition knowledge of interviewees. 

Interviewees were aware of the importance of nutrition and the relationship between the food provided and recipients’ health. They said that some recipients had special dietary needs due to diabetes, heart disease, poor oral health, excessive body weight, or drug or alcohol dependency.

Food handling and safety training was rare for staff and not available for volunteers. The exception was DS with premises, such as the aged care nursing home, whose license required that all staff be trained to meet Australian food safety standards. Although no DS received government funding to deliver nutrition education, four had nutrition programs adapted from the FOODCents^©^ budgeting, purchasing, and cooking skills development program [35,36] and several expressed interest in nutrition and budgeting training.

#### 3.2.5. Influence of Government Policies or Legislation

DS interviewees said that state or federal government food-related policies were limited and had little or no day to day influence on the operations of DS. However, local government parking and public nuisance by-laws negatively impacted mobile services by limiting the locations where they could operate. DS referred to “Good Samaritan” legislation that protected them from liability for any unintended consequences of their activity and they were aware that food safety regulations did not currently apply to them as they did not sell food.

When asked about what was needed to improve DS, they wanted more meat, fish and dairy, fresh fruit and vegetables, sliced bread, facilities and equipment (larger kitchen, more vans, refrigerators, commercial bread slicers), the capacity to extend their weekend outreach services, including a bus with more refrigeration and a barbecue onboard, and the resources and capacity to serve a three-course seated meal.

#### 3.2.6. Overall Barriers to CFS Meeting Demand for Nutritious Food

There is no overarching system or policies directing the CFS in inner-city Perth. Many interviewees did not know or communicate with other services. Even though IS said they preferred to supply nutritious foods to DS, they all received and passed-on donated food (for example, cakes, pastries, soft drinks and other unhealthy foods and drinks, particularly those from bakeries and supermarkets). IS said that their reliance on donated food was the main influence on the food they supplied to DS; they cannot predict their food inventory and are often not able to meet DS needs. Foodbank WA and Food Rescue WA had more consistent donations, but OzHarvest said that donations were unreliable in the type and quantity of food and that they never know what they will get from donors and remarked on the challenges this presents. The IS recommended that food donors be educated regarding the importance of healthy food. 

While one DS said they preferred to provide fruit juice and not carbonated sugar-sweetened beverages, another said that “food is food” and they did not hesitate to provide any food. Mobile services purchased and distributed meat pies because they were convenient, easy to prepare and recipients preferred them. One DS interviewee said it was important to give people the occasional “treat” food.

## 4. Discussion

The findings of this study need to be considered in the context of neo-liberal market economies, where a decline in social welfare creates conditions for individual insecurity and stress [37]. Efforts to reduce pressure on government spending has seen a rise in third sector or voluntary organisations involved in efforts to address complex human problems such as food insecurity [38,39]. The study findings show the CFS in inner-city Perth is complex, with disparate organisations working in an uncoordinated way in difficult conditions. A significant number of operational challenges face both IS and DS, limiting their ability to deliver nutritious or appropriate food relief to recipients. These include the increasing demand and long-term nature of food insecurity (with some models consisting of 1–2 days of emergency relief); their human resource capacity being heavily reliant on volunteers; declining and/or unreliable financial support; an unreliable and inconsistent food supply based primarily on donated or rescued waste food; no food safety or nutrition policy or regulatory framework; and limited nutrition capacity and expertise. No organisation has a standalone ratified nutrition policy supporting the regular acquisition of a nutrition-focused food supply. Disconnected and incoherent policy making is a key challenge in global food systems [40] but equally applies to charitable food systems. In CFS, the policy disconnect and incoherence occurs because decisions are being made in different spaces by diverse policy actors, e.g., government departments, IS, DS, food donors and referring social welfare agencies, which serve diverse interests. Good policy requires a clear understanding of what we want to achieve; so, in this example, is it reducing food waste or reducing food poverty?

DS have offered food relief for up to 125 years demonstrating both the long-term nature of food insecurity and their commitment to providing food to people in need. IS, a more recent addition to the CFS, bring a sophisticated business proposition to food rescue, particularly Foodbank WA based on its organisational capacity. The intent of DS was that they were aspiring to achieve what Hamm and Bellows (2003) call community food security, “a situation in which all community residents obtain a safe, culturally acceptable, nutritionally adequate diet through a sustainable food system that maximizes community self-reliance, social justice, and democratic decision-making” (p. 37) [41,42], whereas IS focused on redistributing food waste without the emphasis on empowering people out of food security.

The inner-city Perth CFS exhibit all three failures (market, government and voluntary) of welfarism described 30 years ago by Lester Salamon [28]. The inability of some citizens to be able to afford to purchase sufficient food in a wealthy country such as Australia is evidence of market failure. The size, scope, expansion and longevity of the food relief sector is a marker of both Government social policy failure in terms of living and income standards and dignified food access [20]. It is also a government failure in terms of the Nation states obligation with respect to the right to food for all citizens—namely to respect, protect and fulfill. Evidence of the four voluntary failures of the non-profit sector includes the following.

### 4.1. Philanthropic Insufficiency

The increasing demand for food relief and the failure of the CFS to meet that demand is evidence of philanthropic insufficiency. The unreliable and inadequate funding, workforce, food and limited facilities undermine the capacity to support the provision of appropriate food relief. The length of CFS organisations’ operation supports the findings of a 2012 review of the WA Emergency Relief secto, which concluded that it will always exist as a safety net, arguing that government should make it a program within a legitimate framework with a funding process [25]. The current findings suggest insufficient government policy, in particular, to assist the CFS to provide safe, reliable and nutritious food to recipients who represent a population sub-group vulnerable to poor health.

CFS relied heavily on corporate, philanthropic and individual donors in inner-city Perth and Victoria due to the limited and unreliable government funding [20]. Charities contribute a significant proportion of their income to CFS; for example, food accounted for 62% of the assistance provided by St Vincent de Paul Inc. in WA in 2017, an expenditure of AUD$1.15 million [43]. During the same period, the Victorian arm provided AUD$14.9 million in material aid, of which 71% was food-related (47% as food vouchers or gift cards) [44]. Getting food to recipients is the focus of effort, leaving little if any time for evaluation of activities for effectiveness or efficiencies. There is some concern that charitable donations have been declining in WA; for example, in 2014–2015 the Salvation Army’s income was AUD$700,000 short of its donation target [45]. 

Interestingly the sentiment expressed by interviewees in this study was that there was enough food, but that it was not able to be distributed effectively, and that resources were wasted. As far back as the 1890s, there was a recommendation that charities in a local area should coordinate to achieve their common purpose and there is limited evidence of co-ordination in our study [28].

### 4.2. Philanthropic Particularism

DS who focused their donations towards particular subgroups—for example, only providing sandwiches for homeless young people on the street or food for people with HIV—were evident. Particularism also extended to local government, who prefer “pop-up” commercial food trucks catering for transient community events. As a consequence of this example, DS mobile vans and their recipients were regularly asked to move on. 

### 4.3. Philanthropic Paternalism

There was a discord between DS organisations’ intent to relieve hunger and promote pathways out of food insecurity and the current practice. The unreliable food supply and lack of nutrition policy meant DS were unlikely to refuse food or beverages of poor nutritional value. The lack of a nutrition policy in Australia is well known [12,26]. Yet, interviewees did not feel that complying with a nutrition policy would limit their food acquisition, consistent with previous research that has found no detriment to services with the establishment of nutrition policy that increases nutritious food acquisition and provision [13,14,46]. A more formal and professional approach is needed to ensure recipients of food relief needs are met.

### 4.4. Philanthropic Amateurism

Philanthropic amateurism was demonstrated by a lack of food service and/or nutrition training, in part due to the reliance on a “well-meaning but largely untrained workforce to deliver the service”. As in other Australian cities, volunteers underpin the CFS workforce in inner-city Perth, suggesting a need for specific volunteer and staff training [20]. Volunteers were mainly retired older people, students, or from large corporate organisations who gift their workforce’s time for community service. Although there are acknowledged benefits of reciprocity, there are also challenges relating to the health, financial resources and the preferred contribution of the workforce [47,48]. Complying with Australian standards for volunteering (matching roles to skills, supporting and developing the workforce, protecting their safety and wellbeing, recognizing contribution and continuously improving) [24] is difficult.

The lack of supportive government policy and legislation contributed to the CFS voluntary failure. Mobile food services were vulnerable to local government regulations who can withdraw permission to operate at their discretion. Interviewees described numerous examples of services being moved on due to construction, festivals, parking restrictions, or conflict. Locating DS indoors would alleviate these problems and provide dignified seated meal services and socialisation, critical for people who are socially isolated [24]. This would also facilitate contact with additional services (e.g., health, accommodation, or supports for employment readiness). The FreshPlace model is an example of this type of integrated service which provides both food and assists pathways out of food insecurity [49].

Maintaining food safety along the logistics supply chain is likely to be difficult in the current CFS given the reliance on rescuing perishable waste food that may be past or close to expiry, and untrained volunteers handling food. Ensuring food handling and safety practices could protect this high-risk population sub-group against foodborne illness. Food safety legislation was designed to reduce the public health risk to the individual, yet the Western Australian Civil Liabilities Act 2002 Volunteers and Food and other Donors Act “protects persons who donate food or grocery products from incurring civil liability for personal injury resulting from the consumption of that food or the use of those grocery products, and for related purposes.” [50] (p. 1).

CFS-focussed hospitality training would build the confidence and efficiency of the workforce in food service management, food procurement, menu planning, food preparation, occupational health and safety, food safety, and nutrition.

### 4.5. Equity, Effectiveness and Efficiency

This current study also provides evidence of inadequacy and the corresponding need for action to address all three areas of the ‘iron triangle of hunger relief’ described by Sengul Orgut et al. (2017) [51]. The three areas are equity (serving the needs of the recipient fairly in regard to both the quantity and quality or type of food received), effectiveness (the ability to meet the needs of the food insecure recipient), and efficiency (cost of resources needed to collect, manage, store and distribute donated food). Each dimension is in turn affected by supply (uncertain monetary supply, donations, and perishability), distribution (uncertain demand) and capacity factors (physical storage, transportation, workforce, and budget). Inefficient food redistribution is exacerbated by a lack of communication between CFS organisations and concerns were raised about overlapping or even competing services, and apparent lack of coordination.

### 4.6. Strategic Partnerships—The Way Forward

Based on the findings of this organisational audit, there are seven recommendations to guide action to improve the capacity of the CFS to provide a food service that meet the needs of its recipients. The recommendations are ranked in order of priority and given the similarities of the CFS in other Australian States and Territories, we believe they have national applicability.

1. Government-led framework with strategic coordinated partnerships with policy, licensing and funding supports

Streamlining the coordination and collaboration to reduce duplication and provide a better-quality service is recommended. The voluntary sector’s weaknesses correspond to government strengths, and vice versa. In the case of the CFS, all levels of government (national, state and local) could partner with the DS and IS. Each level of government has a different imperative; for example, local area health plans are required to address significant health needs of the community, statewide departments act as system managers to set policy priority and conduct monitoring and surveillance activities, and the Federal government sets national standards, develops quality improvement schemes and is responsible for emergency response and social welfare decision making. Funding opportunities and decisions could then occur across all three levels of government and with all partners. Special care would need to be taken to ensure this is achieved without the loss of autonomy or flexibility for the CFS to meet recipient’s needs.

2. Refocus, resource and prioritise the requirements for a nutrition-focussed CFS.

Planned menus are integral to the provision of a safe, nutritious and appropriate food service, and a reliable food supply is essential. The scope and nature of the IS suggest that the timing is right for them to focus on nutritious food acquisition with a formalized policy, such as that achieved with nutrition-focussed food banking [13]. Government can support the policy development and implementation through appropriate licensing and/or regulation to address any food safety or nutrition risk and sustain the change with additional resources.

3. Establish CFS principles and standards for appropriate food service needs.

The duty of care is described and controls (policy, licensing, legislation, accreditation and/or training) are implemented in other areas where foodservices are offered to vulnerable population sub-groups; for example, for children (in childcare centers, schools, or day care [31,32]) or people in custodial facilities, aged care facilities, or hospitals where recipients are reliant of the food provided to meet their welfare needs. At a local government level, compliance with food safety regulations for events such as festivals, music concerts are tightly controlled but do not apply for CFS. 

Local government is currently considering licensing mobile CFS, including standards that translate nutrition needs across the continuum of care into the types and amounts of foods that should be acquired and supplied to meet CFS recipients’ needs in a timely, cost-effective way to improve CFS. Work needs to be undertaken to determine both the content and ‘format’ (how the food is distributed, utilised and mechanisms for social inclusion) as was undertaken in Belgium [52]. A realistic individual assessment of the length of time the DS is needed should be included in the assessment.

Food safety training should be a mandatory requirement for all CFS workers handling food. As with retail food business, measures should be taken to ensure food safety. The large and changeable volunteer workforce and limited funding may hamper training opportunities; however, given the types of perishable foods distributed, particularly eggs, meat, prepared meals, sushi, there is likely an increased the risk of food poisoning in a system without a food safety and handling framework.

4. Explore options to increase the sufficiency and efficiency of the food supply

Efficiencies are needed in both the distribution of food from IS to DS to recipients and its transformation into appropriate forms suitable for different food service models. With coordination, many options are available to improve food supply logistics and efficiencies. Technology-based online inventories of donated food used to improve food distribution efficiencies in other developed countries [53,54,55] are not used in Perth. These systems could improve efficiencies by signposting food availability earlier based on “use-by” or “best-by” dates; increasing donations of perishable items; assisting small CFS with limited food storage and with disaster relief emergency responses for food at short notice and in large quantities.

For a sustainable CFS system, government and the commercial and voluntary sector should consider the following: what are the cost benefits of redistributing food waste from the retail sector? Who pays, and what are the costs at each stage of the supply chain, including the food service end? Are there other preferred options? Improving efficiencies may also lead to resources being freed up to re-direct to other priorities; for example, providing meal services on weekends and during holiday periods where they are currently not provided.

5. Training and development of the CFS workforce is needed

Develop and provide CFS workforce training to enable delivery of services to meet their organisational intent and recipient’s needs, framed as providing community food security. Cost-effective options should be investigated such as using the massive open online course (MOOC) platform, which enables flexible participation and uses a contemporary educational design to show case studies and provide opportunities for interactive learning and has been shown to be effective [56,57]. The “Developing Food Bank Nutrition Policy to Procure Healthful Foods” (Canvas.net) MOOC for food banks provides a precedent [58]. Local government, peak volunteering bodies, or hospitality training colleges or universities could consider offering gratis training for CFS staff and volunteers.

6. Develop a CFS measurement system monitoring demand, distribution, impact and economic benefit

The CFS works to provide community food security, yet currently measures their impact in terms of kilograms of food rescued or meals provided. Consistent system-wide measures would enable all players in the CFS to articulate its value in terms of achieving community food security. Specific cross-discipline higher degree research should be a university research and government funding priority.

7. Reorient the CFS to create pathways to build sustained food insecurity for recipients

The values of DS organisations suggest that the aim of the CFS approach should be to ensure “community food security” which focusses on local sustainable solutions to ensure ongoing food security rather than just providing short-term food relief. Inherent in any CFS response is the need for higher degrees of citizen empowerment and food democracy, not evident in Perth CFS recipient views [24]. Reducing food insecurity is also an internationally acknowledged government public health priority. Placing people’s lived experiences at the centre of decision delivers better integrated policy solutions and effective pathways out of food insecurity [40]. The lack of uptake of innovative social enterprises as a response to food insecurity in Australia has been attributed to the resistance from dominant commercial players and restrictive government legislation [20]. There is a need to work with these actors to support the development and trialing of alternative models to address the market, government and voluntary failures that exist in the current CFS.

### 4.7. Strengths, Limitations and Further Research

This study is the first comprehensive examination of the scope and operational capacity of the charitable food sector in inner-city Perth and provides a detailed picture of the workings of the sector in an Australian capital city. Specific information on corporate donors and funding was not provided due to confidentiality; however, additional information was sought from financial reports. Service delivery achievements and shortfalls were inconsistently expressed across organisations; for example, millions of meals served versus tonnes of food waste diverted from landfill and numbers of people provided meals. Turn away rates due to short falls in food supply were not available but would assist in the assessment of the effectiveness of the CFS. The findings of this study are limited to the CFS provided in inner-city Perth and provide only a snapshot at a point in time; however, they are likely to be relevant to other the inner-city precincts of Australian and international capital cities with a welfare safety net.

Further research is needed to quantify the types, amount and form of food supplied and the environment in which it is delivered. This will help to determine the suitability and capacity of CFS to meet of the needs of their recipients in terms of food security, nutrition status, and social inclusion. Current decision-making is divorced from lived experience. Decision makers are crafting solutions devoid of an understanding of those who are affected by the problem. Further research validating people lived experience of food insecurity and trialing new responses which offer pathways out is a priority.

Further research recommendations include an economic cost-benefit analysis of the efficiencies of the CFS; further exploration of the government (federal, state and local) and private sector roles in the CFS; and the development and piloting of other models of food relief with an emphasis on social inclusion and pathways out of food insecurity.

## 5. Conclusions

This research is a timely contribution that shines a light on the NP sector as it struggles to cope with the chronicity of embedded food insecurity, the ad hoc nature of donated food, declining funding and resource constraints. The lack of formalised nutrition policy and training is likely to hinder the acquisition and provision of nutritious and appropriate food relief for people vulnerable to food insecurity. Coordination, reliable funding and food acquisition, food handling and nutrition policy and training and volunteer support is needed to build the capacity of the sector. The findings suggest a CFS at breaking point and highlight the urgent need for debate and investigation of other models to better address food insecurity.

## Figures and Tables

**Table 1:** Semi-structured interview schedule.

Topics Covered	Indirect Services	Direct Services
Organisational values, length of operation, funding sources	Asked	Asked
Food service models/types, location, timing, description of recipients	Asked	Asked
Workforce profile including volunteers and training	Asked	Asked
Food storage capacity	Asked	Asked
Nutrition and food safety training, policy and practices	Asked	Asked
Sources of foods	Asked	Asked
Food transport (for food received and distributed)	Asked	Asked
Perception of donors influence on charitable food services (CFS)	Asked	Asked
Impact of government actions on CFS	Asked	Asked
Preferences for specific foods	Asked	Asked
Challenges and opportunities to increasing nutritious food	Asked	Asked
Agencies receiving food, quantities and recipients	Asked	Not asked

**Table 2:** Snapshot of the types and extent of direct food service in inner-city Perth, January 2015.

Funding	Food Source	Food Model	Days of Operation							Serve	Staff	
			M	T	W	T		S	Su	/wk	Paid	Vol
G (CP)	D	S, S/W (MS)	√	√	√	√	√	√	√	500	4	565
CH	D	Parcels		√			√			-		
CH	D	BBQ Monthly								-		
G (CP, H)	P	Kitchen	√	√	√	√	√	√	√	-		
D	D (f&P), B, R	TA noon	√	√	√	√	√	√		1250	5	200
CH	D (f&P)	BF (SM)	√	√	√					30–45		
CH	D (f&P)	Lunch (SM)	√	√	√		√			30–45		
CH	D (f&P)	D (SM)				√	√	√		120		
D, CH	D (f&P), B, R	BF (SM)	√	√	√	√				150	6	
D, CH	D (f&P), B, R	Food Any	√	√	√	√	√			-		
D, CH	D (f&P), B, R	Lunch					√			-		
D, CH	D (f&P), B, R	Parcel	√	√	√	√	√			-		
D, CH	D (f&$), B, R	Voucher	√	√	√	√	√			-		
Lottery	D	Parcel	√	√	√	√	√			5		
Lottery	D	Voucher	√	√	√	√	√			-		
G (CP)	D, R	MT	√	√	√	√	√			650	4	40
CH	D (f&$)	S Kitchen						√		20–50		
G (CP, H)	B, R	All meals	√	√	√	√	√	√	√	144	4	6
Lottery	D	S/W	√	√	√	√	√			5		
CH	D	Pantry	√	√	√	√	√			75		
CH	D (f&P), B, R	Café	√	√	√	√	√			-	2	153
CH	D (f&P), B, R	Parcel/Pantry	√	√	√	√	√			250		
CH	D (f&P), B, R	Voucher $20	√	√	√	√	√			150		
CH	D (f&P), B, R	S, S/W (Van)	√	√	√	√	√	√	√	350		
CH	D (f&P), B, R	S, S/W (Van)					√	√	√	150		
CH	D (f&P), B, R	All meals	√	√	√	√	√	√	√	600	4	20
CH, G CP	D (f&P), B, R	Drop-in (SM)	√	√	√	√	√			500	1	1
Total										5029	30	985

√: yes; &: and; G: government; CP: Department of Child Protection; CH: church; H: Department of Health; TA: take-away, P: purchased; D: donated; f: food; $: money; B: bank; R: rescue; SM: seated meal; UCW: Uniting Care West; BF: breakfast; MT: morning tea; L: lunch; D: dinner; S: soup; S/W: sandwich; M: Monday; T: Tuesday; W: Wednesday; T: Thursday; F: Friday; S: Saturday; Su: Sunday; wk: week; Fed: Federal; Vol: volunteers.

## Supplementary Materials

The following are available online at http://www.mdpi.com/1660-4601/15/6/1249/s1, Table S1: Characteristics of Charitable Food System Indirect Service organisations servicing or located in the inner-city Perth.
