# Supplementary material for: Charitable Food Systems’ Capacity to Address Food Insecurity: An Australian Capital City Audit
Source: Int J Environ Res Public Health. 2018 Jun 12;15(6):1249. doi: 10.3390/ijerph15061249 (PMC6025598; doi:10.3390/ijerph15061249)
Supplement: Supplementary file 1 [file ijerph-15-01249-s001.pdf]

Supplementary Table S1. Characteristics of Charitable Food System Indirect Service organisations servicing or located in the inner-city Perth

Name	Foodbank WA	OzHarvest [38]	Food Rescue WA [39] and Second Bite
Type of organisation	Food banking	Food rescue	Food rescue
Purpose	"*…fight hunger in Western Australia by providing **quality food to people in need** and by delivering food education that promotes healthy eating*" [36] (p2).	To stop food going to waste…collect ***quality surplus food*** from commercial outlets and deliver it directly to charities supporting ***people in need***.	"*alleviate hunger in WA by rescuing perishable, fresh and nutritious food from cafes, caters, supermarkets and wholesalers and delivering it to disadvantage and vulnerable people*" [37] (p 26)
Food distribution	Collect from large retailers, farmers and bank or prepare it for *DS to collect*.	Collect food from commercial outlets and deliver it to DS.	Collect food from supermarkets, sort and repack it and deliver it to DS.
Facilities	Large custom built premises (commercial kitchen, dry and cold storage (refrigerator and freezer))	No sorting or storage capacity so distribute immediately	Refrigerated and freezer storage space premises
Location	27 km from CBD at the airport	Store vans inner-city	10 km from CBD
Transport	Fleet of trucks	Two vans	Three vans (two cargo carts)
Length of operation	21 years	2 years (2014)	6 years (2010)
Nutrition capacity			
Accept unhealthy	Yes	Yes	No
Nutrition policy	No	No	No
Nutrition training	None inner city Perth	2 Sessions in last year	None
Food safety	Train food preparation staff	Do not accept unsafe food	Do not accept unsafe food
Food safety audits	Regular government food safety audits	Not required under Food Act	Not required under Food Act
Funding	2017 [37] (AUD$)		
Type			Philanthropy
Statewide Total	8.63 million		
Handling fee/kg food	3.78 million		
Grants	2.44 million		
Sponsorship/donors	1.29 million		
Expenditure on food	772,385 (8.95%)		
Healthy Food For All	592,018 (6.86%)		
Funding source	Corporate, private donations and government grants	Corporate donors	

Supplementary Table S1. Characteristics of Charitable Food System Indirect Service organisations servicing or located in the inner-city Perth

Workforce			
Paid staff (n)	50 FTE	6	2
Volunteers	192 week plus 785 corporate /year	150 (80 drivers)	100
Staff:volunteer ratio	1:45	1:25	1:50
Nutrition workforce	(n)	(n)	(n)
Nutritionists	10 (not inner-city)	1 nutrition coordinator (casual)	nil
Health promotion	2 (not inner-city)	nil	nil
Student placements	Yes, nutrition.	Yes, hospitality and nutrition.	nil
Chef	1	1 (casual)	nil
Nutrition focus	Yes, Key Staples Program	?	Yes
Accept unhealthy food	Yes	Yes	Yes
Food			
Amount / year(kg)	2,800,000	348,627	478,000
Meal equivalents	5,602,080 meals (WA)	na	
Recipients per week	11,800 + 22,864 emergency meals (WA)	na	5,500
Fresh fruit, veg (kg)	70,000	na	99%
Distribute to	510 DS and 432 schools in 2014/15.	30-45 DS inner-city Perth 84 DS	69 DS
Procurement method	Partnerships donations with the local food and grocery industry	Partnerships donations, collect from inner city food businesses	Partnerships donations, collect from inner city food businesses
Food retrieval intent	To reduce food waste and purchase	To reduce food waste	To reduce food waste
Description	"*out of specification, close to its use by date, incorrect labelling, damaged packaging, as well as excess stock or deleted lines.*" [37] (pg 8).	Collect perishable food from retailers and venues such as cafes, restaurants, supermarkets (75% of food), caterers and bakeries.	Collect perishable food from retailers and venues such as cafes, restaurants, supermarkets, caterers and bakeries.
Packaged	74%	None	1%
Perishable	26%	All, fruit and vegetables, frozen meals, sushi, prepared sandwiches and mini-quiches.	99% fresh fruit and vegetables, prepared sandwiches, rolls, wraps and pastries
